# Early-stage COVID-19 pandemic observations on pulmonary embolism using nationwide multi-institutional data harvesting

**DOI:** 10.1038/s41746-022-00653-2

**Published:** 2022-08-19

**Authors:** Axel Wismüller, Adora M. DSouza, Anas Z. Abidin, M. Ali Vosoughi, Christopher Gange, Isabel O. Cortopassi, Gracijela Bozovic, Alexander A. Bankier, Kiran Batra, Yosef Chodakiewitz, Yin Xi, Christopher T. Whitlow, Janardhana Ponnatapura, Gary J. Wendt, Eric P. Weinberg, Larry Stockmaster, David A. Shrier, Min Chul Shin, Roshan Modi, Hao Steven Lo, Seth Kligerman, Aws Hamid, Lewis D. Hahn, Glenn M. Garcia, Jonathan H. Chung, Talissa Altes, Suhny Abbara, Anna S. Bader

**Affiliations:** 1 Department of Imaging Sciences, University of Rochester Medical Center, Rochester, NY USA; 2 Department of Biomedical Engineering, University of Rochester Medical Center, Rochester, NY USA; 3 Department of Electrical and Computer Engineering, University of Rochester, Rochester, NY USA; 4 Faculty of Medicine, Ludwig Maximilian University of Munich, Munich, Germany; 5 Department of Radiology & Biomedical Sciences, Yale University School of Medicine, New Haven, CT USA; 6 Department of Radiology, Mayo Clinic College of Medicine and Science, Jacksonville, FL USA; 7 Department of Radiology, University of Massachusetts Chan Medical School, Worcester, MA USA; 8 Department of Radiology, University of Texas, Southwestern Medical Center, Dallas, TX USA; 9 Department of Imaging, S. Mark Taper Foundation Imaging Center, Cedars-Sinai Medical Center, Los Angeles, CA USA; 10 Department of Radiology, Wake Forest School of Medicine, Winston-Salem, NC USA; 11 Department of Radiology, University of Wisconsin, Madison, WI USA; 12 Department of Radiology, Christiana Care Health System, Newark, DE USA; 13 Department of Radiology, University of California, San Diego, San Diego, CA USA; 14 Emory University School of Medicine, Department of Radiology and Imaging Sciences, Atlanta, GA USA; 15 University of Texas, Medical Branch, Galveston, TX USA; 16 Department of Radiology, University of Chicago, Chicago, IL USA; 17 University of Missouri-Columbia, Columbia, SC USA

**Keywords:** Viral infection, Diagnosis

## Abstract

We introduce a multi-institutional data harvesting (MIDH) method for longitudinal observation of medical imaging utilization and reporting. By tracking *both* large-scale utilization *and* clinical imaging results data, the MIDH approach is targeted at measuring surrogates for important disease-related observational quantities over time. To quantitatively investigate its clinical applicability, we performed a retrospective multi-institutional study encompassing 13 healthcare systems throughout the United States before and after the 2020 COVID-19 pandemic. Using repurposed software infrastructure of a commercial AI-based image analysis service, we harvested data on medical imaging service requests and radiology reports for 40,037 computed tomography pulmonary angiograms (CTPA) to evaluate for pulmonary embolism (PE). Specifically, we compared two 70-day observational periods, namely (i) a pre-pandemic control period from 11/25/2019 through 2/2/2020, and (ii) a period during the early COVID-19 pandemic from 3/8/2020 through 5/16/2020. Natural language processing (NLP) on final radiology reports served as the ground truth for identifying positive PE cases, where we found an NLP accuracy of 98% for classifying radiology reports as positive or negative for PE based on a manual review of 2,400 radiology reports. Fewer CTPA exams were performed during the early COVID-19 pandemic than during the pre-pandemic period (9806 vs. 12,106). However, the PE positivity rate was significantly higher (11.6 vs. 9.9%, *p* < 10^−4^) with an excess of 92 PE cases during the early COVID-19 outbreak, i.e., ~1.3 daily PE cases more than statistically expected. Our results suggest that MIDH can contribute value as an exploratory tool, aiming at a better understanding of pandemic-related effects on healthcare.

## Introduction

Coronavirus disease 2019 (COVID-19) has had a profound impact on medical imaging^1,2^, and recovery from the pandemic will require understanding these effects in order to plan for the future^3^. Studies have demonstrated the usefulness of temporal tracking of radiology utilization data, which can guide institutions through these unusual circumstances^1–3^. Here, we introduce an approach that not only tracks such data from the viewpoint of imaging services utilization but creates an opportunity for gaining insights into pathologic clinical findings, such as for investigating the prevalence of observed disease entities and specific disease-related complications.

Establishing trends related to computed tomography pulmonary angiography (CTPA) during the COVID-19 pandemic is particularly meaningful in this context. CTPA is commonly used to evaluate the pulmonary arteries for the presence of pulmonary embolism (PE) and can be performed on inpatients, outpatients, and in the emergency department. In the early phase of the COVID-19 pandemic, there were several competing factors affecting PE study utilization. On one hand, public health lockdowns and patient avoidance of medical facilities was decreasing the number of studies performed, even when those studies were indicated, delaying diagnosis of many conditions^4,5^. The COVID-19 pandemic affected the rates of all types of imaging, with a decline in most types of studies, most pronounced among outpatient imaging^2,6^. Meanwhile, there was growing evidence that COVID-19 infection was a significant risk factor for developing PE. Early in the pandemic, several autopsy case series suggested that many patients who died from COVID-19 had thromboembolic disease at autopsy^7–10^, and other cases suggested that it was the cause of death^11^. Subsequently, studies showed that COVID-19 infection causes an inflammatory cascade that is prothrombotic^12–16^, and in many cases, can lead to in situ thrombosis in the pulmonary arteries^17^. Clinical case studies began to confirm this trend with several studies showing an increased incidence of PE in patients hospitalized with COVID-19 (between 8 and 16%)^18–21^, and specifically among patients in intensive care units (up to 20%)^18,22,23^. Radiology case reviews also reported high rates of PE-positive CTPA studies, ranging from 17 to 50%^24–32^. Multiple clinical trials were initiated attempting to optimize anticoagulation of COVID-19 patients, but it was not until June 2020 that expert guidelines were published^33^. By the summer of 2020, growing awareness among clinicians that PE was a serious complication of COVID-19 was changing CTPA ordering patterns once more.

Due to changes in overall healthcare utilization, evolving data on the association of thromboembolic events and COVID-19, and potential changes in disease prevalence, it was unknown how the pandemic would affect PE prevalence. Neither was it known whether CTPA ordering patterns or the rate of observed PE-positive CTPA studies might be affected by the pandemic.

Here, when looking for patterns in data over time, studies from single institutions face the challenge of small cohorts resulting in low statistical power. To address this challenge, we introduce a multi-institutional data harvesting (MIDH) approach as a method for establishing important disease-related trends over time, such as the observed prevalence of PE in CTPA studies during the COVID-19 pandemic. The MIDH approach tracks both imaging utilization and radiology report findings. At many hospitals, including all institutions participating in this study, artificial intelligence (AI) image analysis services are being used to screen medical imaging exams, expedite workflow, and improve radiologists’ diagnostic accuracy^34,35^. Prioritization software originally developed for orchestrating the data workflow for such AI-based image analysis services can be repurposed to access and collect data about radiology study utilization and reported findings. Specifically, while using such repurposed IT infrastructure for case identification and data collection only, we did not use the results of AI-based image analysis as the ground truth for identifying PE-positive CTPA cases but performed natural language processing (NLP) on final radiology reports instead. To establish the validity of this approach, we examined the accuracy of NLP^36^ for classifying radiology reports as positive or negative for PE in a multi-institutional NLP validation trial. This is in line with recent studies that have utilized NLP for extracting information regarding a wide spectrum of medical conditions, ranging from COVID-19-related respiratory illness^36^ to osteoporosis and fractures, with improved performance compared to manual review^37^.

Our overall goal for conducting this work was to demonstrate the feasibility of the proposed MIDH approach by investigating the effect of the COVID-19 pandemic on CTPA case volumes and observed prevalence of PE within a retrospective study encompassing aggregated data from 13 healthcare systems throughout the United States.

## Results

### Patient characteristics

A total of 40,037 CTPA examinations were recorded in the time period from 11/1/2019 through 6/30/2020 from all 13 participating US healthcare systems. Of those, 147 were excluded based on known age below 18 years; 4191 patients with unknown age were included as very few were likely to be children. Of the remaining exams, a total of 21,912 cases were performed within two specific 70-day observational periods, with 12,106 cases within a pre-COVID-19 observation period and 9806 cases during the early pandemic outbreak period. The median age was 59 years (interquartile range (IQR) 45–70 years) among patients with known age, with 56% female patients. Overall, 58% of patients were imaged in emergency departments, 28% were inpatients, and 10% were outpatients; the remainder was unknown or from specific other departments, such as obstetrics. The patient data stratified by observational periods is summarized in Table 1, demonstrating comparable patient populations in each group.

**Table 1:** Patient demographics.

	Pre-COVID (*n* = 12,106)	Early COVID (*n* = 9806)	Overall (*n* = 21912)	*p* value
Age, years, median (IQR)^*^	60 (46–71)	58 (43–69)	59 (45–70)	<0.0001
Female, *n* (%)	6914 (57.1)	5335 (54.4)	12,249 (55.9)	<0.001
Patient location, *n* (%)				<0.001
Emergency Department	7082 (58.5)	5718 (58.3)	12,800 (58.4)	
Inpatient	3298 (27.2)	2844 (29.0)	6142 (28.0)	
Outpatient	1300 (10.7)	904 (9.2)	2204 (10.0)	

*Only patients with known age greater than or equal to 18 years are included in this analysis.

### CTPA utilization and positivity rates

**Fig. 1:**
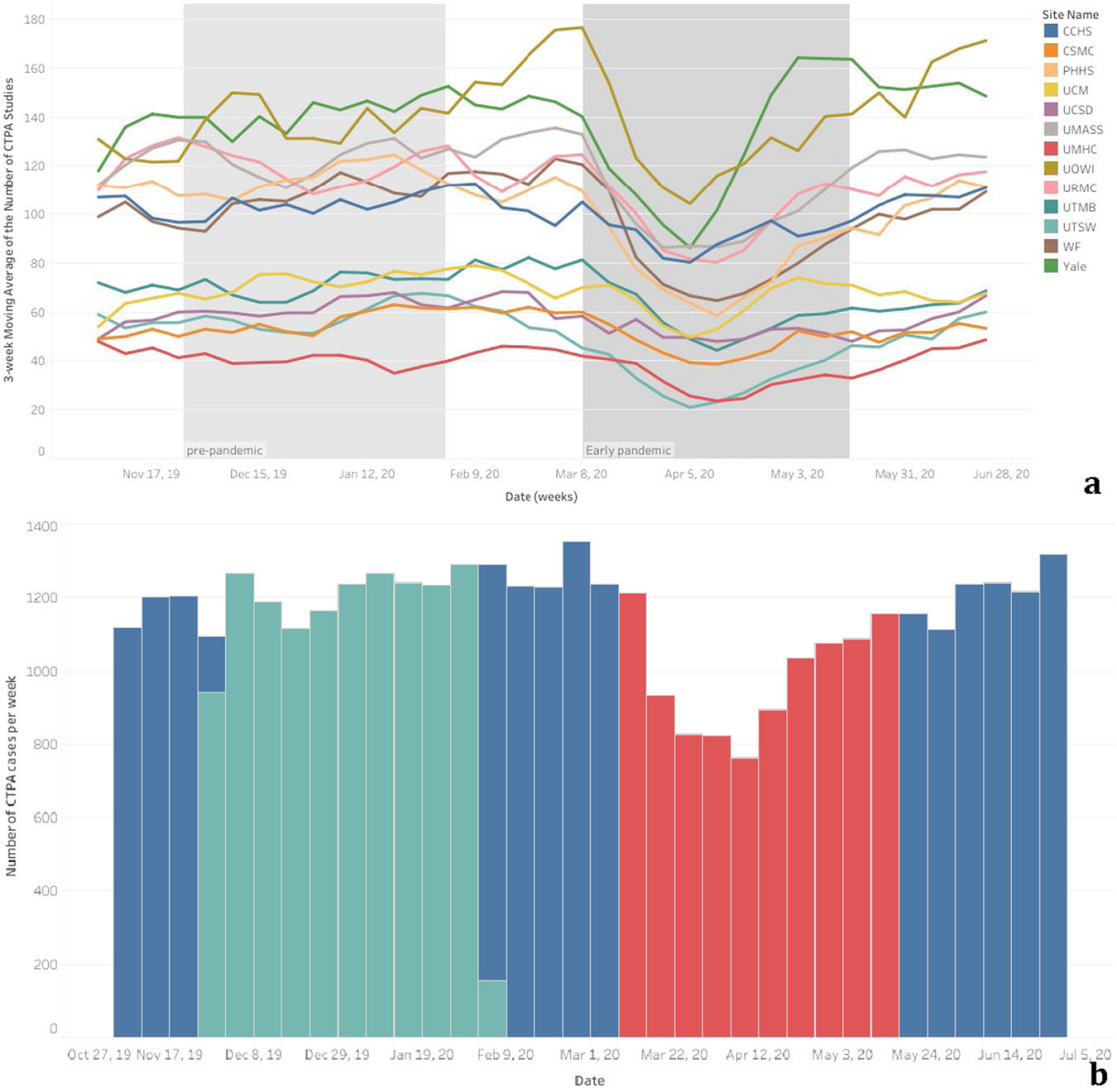
Weekly CTPA exams performed. Total weekly number of CTPA exams performed at each institution (**a**) and across all of the institutions combined (**b**), demonstrating an overall decrease in the early pandemic period (red), compared to the pre-pandemic period (green).

The daily average number of CTPA cases was 174 in the pre-COVID-19 period and 140 in the early COVID-19 period. A decrease in the average number of CTPA exams at the beginning of the COVID-19 outbreak is clearly seen for all institutions leading to an overall decrease in the weekly CTPA numbers for all the institutions combined (Fig. 1).

**Fig. 2:**
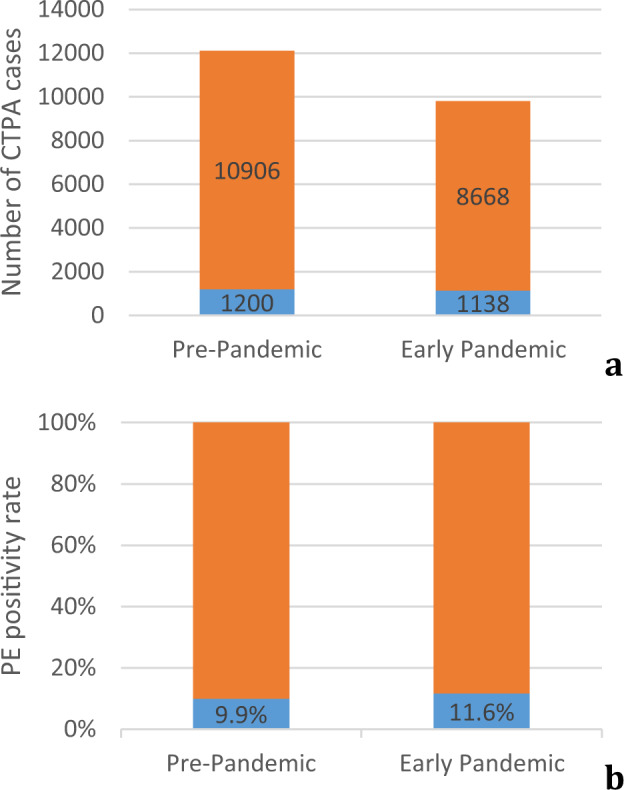
Total acquired CTPA scans and PE positivity rates. Total numbers of acquired CTPA scans (**a**) during the two observation periods demonstrate a clear decrease in the total number of studies performed. Simultaneously, the prevalence of positive PE cases (blue bar) increased in the early COVID-19 period (**b**). For a detailed account of institution-specific data, please see Supplementary Fig. 3 of the Supplemental Material.

**Fig. 3:**
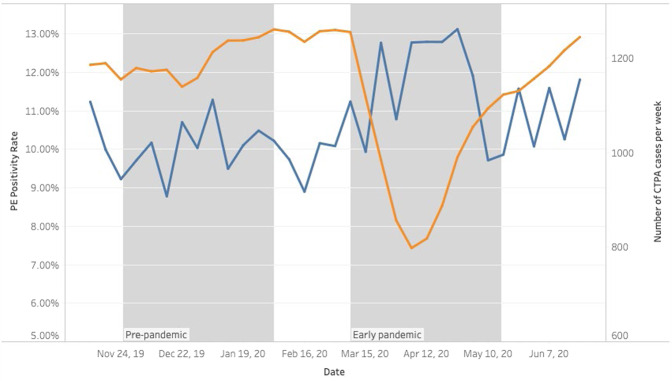
Changes in CTPA utilization and PE positivity rate over time. Superimposed results demonstrate a drop in the centered 3-week moving average of weekly total CTPA exams performed (orange curve) with a simultaneous increase in PE positivity rates (blue curve).

During the pre-COVID-19 period, 1200/12,106 (9.9%) CTPA cases were positive for PE, while 1138/9806 (11.6%) were positive for PE during the early COVID-19 period (Table 2 and Fig. 2). There is a statistically significant association between the ratio of PE-positive CTPA studies (“PE positivity” rate) and the observational period (χ^2^(1, *N* = 21,912) = 16.29, *p* = 0.0001). Note that, for the 70-day early pandemic observational period, we observed an excess of 92 positive PE cases, or 1.3 additional PE cases per day more than statistically expected. In summary, when compared to the pre-pandemic period, there was an overall decrease in CTPA examinations performed with a simultaneous increase of the PE positivity rate (Fig. 3).

**Table 2:** CTPA results during the two observation periods.

Exam Result	Pre-COVID	Early COVID	Total
PE+	1200 (9.9%) [1292]	1138 (11.6%) [1046]	2338
PE−	10,906 (90.1%) [10814]	8668 (88.4%) [8760]	19,574
Total	12,106	9806	21,912

A positive PE result was more likely during the early COVID period compared to before the pandemic. The contingency table provides the following information: the observed cell totals, column percentages in parentheses, and the expected cell totals in brackets.

**Fig. 4:**
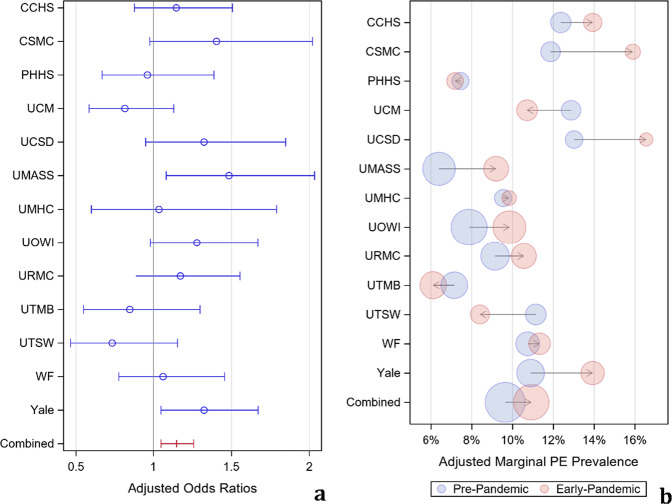
PE positivity and prevalence. Adjusted odds ratios for PE positivity (**a**), with the pre-COVID-19 period as the reference standard, and adjusted marginal estimate for PE prevalence by each site as well as combined (**b**). The sizes of the bubbles are proportional to 1/SE of the % estimates.

When adjusting for gender and patient location, most sites had a positive odds ratio (OR) for positive PE in the early COVID-19 pandemic outbreak period compared to the pre-COVID-19 period, consistent with a higher positivity rate during the early pandemic (Fig. 4). The estimated overall OR was 1.15 (95% CI 1.05–1.26, *p* = 0.04). The adjusted PE positivity rate was 9.6% (8.4–11.1%) in the control period and 10.9% (9.4–12.6%) in the early COVID-19 period. None of the interaction terms between COVID-19 and the covariates were statistically significant at the 0.05 level (*p* = 0.44 (patient location*COVID-19) and *p* = 0.64 (gender*COVID-19)), demonstrating that the effect of patient location and gender on PE positivity was consistent between the two observation periods.

### NLP validation

We performed a multi-institutional NLP validation trial at 12 of the 13 participating healthcare systems. The PE studies from two of the institutions (UT Southwestern Clements University Hospital (CUH) and Parkland Health and Hospital System [PHHS]) were interpreted by one radiology department using identical reporting templates. Therefore, the NLP performance validation was performed only at one of these two institutions (CUH). In total, 1200 PE+ and 1200 PE− by NLP radiology reports were manually reviewed. NLP sensitivity across participating sites was 99.1% (95% CI 97.01–99.01%) and the specificity was 96.4% (95% CI 97.76–99.44%). The overall accuracy was 98% (Table 3). Detailed results from each institution are provided in the Supplementary Material (Supplementary Table 1). Since the site level performance was shown to be adequate at all sites, other commercially available NLP solutions were not pursued.

**Table 3:** A natural language processing (NLP) validation trial using a manual review of 2400 radiology reports was conducted at 12 participating institutions.

	Manual classification		
NLP classification	PE−	PE+	Total
PE−	1198	11	1200
PE+	38	1162	1200
Total	1227	1173	2400

Results demonstrate an overall accuracy of 98%.

## Discussion

In this study, we introduce multi-institutional data harvesting (MIDH) for longitudinal observation of medical imaging utilization and reporting as a digital medicine method for estimating important disease-related observational quantities, such as the observed prevalence of pulmonary embolism on CTPA exams during the early phase of the COVID-19 pandemic outbreak. To accomplish this goal, we combined repurposed software developed for AI-based workflow orchestration and NLP to access and collect data about radiology study utilization and CTPA positivity rates across multiple healthcare systems throughout the United States.

In our multicenter study, we observed a statistically significant increase in the prevalence of PE on CTPA exams during the first wave of the COVID-19 pandemic. Interestingly, this increased prevalence occurred despite a simultaneous decrease in the overall number of CTPA exams performed during that time. This observation was consistent throughout most of the 13 participating health systems. However, four out of 13 individual sites showed the opposite trend with a decreased PE positivity rate in the early COVID-19 period. Possible explanations for this include workflow quality metrics, different patient populations, and geography. For example, at UCM, a quality control measure of preventing imaging overutilization by routine monthly tracking of the PE positivity rates among CTPA studies ordered in the emergency department may have inadvertently lowered the overall rate of studies ordered. Academic referral centers, such as UTSW and UTMB, perform a significant proportion of outpatient imaging exams and have limited capacity emergency departments, possibly leading to a smaller number of patients presenting with emergencies such as PE, and fewer critically ill COVID-19 patients that required referral for CTPA studies once the association was established. Finally, that three out of four of these sites are located in Texas (PHHS, UTSW, and UTMB) may reflect a later temporal peak in the southwestern region that occurred after the time period selected for this study. However, the overall finding is not diluted by these differences.

The variability of PE frequencies among different institutions can not only be explained by their geographic locations, but also by their size, the social structure of their served communities, and specific referral patterns. As such, we were able to observe such differences even in geographically neighboring hospitals affiliated with the same healthcare system. For example, Parkland Hospital (PHHS), a tertiary care institute associated with the UTSW hospital system, is one of the largest in the southwest US and has a substantial underserved population with a broad referral pattern, different emergency admission workflows, and increased sickness severity index, when compared to other UTSW-affiliated hospitals, which may explain the observed differences in PE positivity rates between UTSW and PHHS.

There are several possible explanations for the increased PE prevalence during the early COVID-19 period in many of our institutions. First, our findings may reflect a true increase in case prevalence of PE caused by COVID-19 infection, which is known to induce a prothrombotic state and increases the risk of embolism, both pulmonary and systemic^20,23,38,39^, although some studies have questioned whether the risk is actually higher in hospitalized patients^40^. While coagulopathy can occur in other viral infections, such as H1N1 influenza A^41^, many case reports suggest that the prothrombotic state associated with COVID-19 is even seen in subclinical infections^42,43^, so the presence of the SARS-CoV-2 virus in a population may be enough to raise the risk of PE even in relatively healthy outpatients.

Another possible explanation for our findings is that the subset of patients that were seeking medical care during the pandemic were in an overall more severe clinical condition with a higher pre-test probability and therefore had a higher ratio of patients with PE. This trend has been identified in other conditions. For example, patients with diverticulitis were more likely to present with complicated diseases during the pandemic compared with before COVID-19^44^. Clinician ordering practices likely changed during the pandemic as well. Once case reports, society guidelines, and news stories throughout the early summer of 2020 raised awareness of the increased risk of PE in COVID-19 patients, providers may have begun to order more CTPA studies, which likely explains some of the increase in volumes towards the end of our study period. Another hypothesis is that the sedentary lifestyle many people tended toward during quarantines and public lockdowns may have affected the observed spike in PE prevalence. Secondary analyses are required to weigh the effects of these explanations, and each may hold true for different regions of the US during different time periods.

Our study has several limitations. First, our investigation was based on numbers of reported PE rather than on individually diagnosed PE. Despite the measured high diagnostic accuracy for PE classification based on the NLP validation study, the NLP approach has its limitations, as it will never achieve perfect diagnostic accuracy, regardless of the encouraging validation study results. Additionally, in our current implementation, the NLP classification does not encompass potentially clinically relevant details related to PE types, such as central, peripheral, acute, chronic, multi-focal, etc. Given the verification mechanism inherent to our NLP validation trial study design, with the inclusion of nearly all participating institutions and cross-checking subsets of reports between institutions, we believe that this ground truth surrogate for defining the presence of PE was sufficiently robust for the purposes of the current investigation. In this context, it should be emphasized that our study did *not* use the imaging information of the CTPA studies directly, but solely relied on the radiology report as the “ground truth” for determining the presence or absence of PE. Specifically, we did not investigate the radiologists’ diagnostic accuracy when reporting PE studies, nor did we evaluate the performance of scientific or commercial products for automatically detecting PE in CTPA studies. Although there are future research opportunities linked to such further analyses, these are out of the scope of our current investigation.

We automatically retrieved all CTPA studies dedicated to potentially detecting PE based on radiology procedure description. Therefore, we cannot exclude that a small number of actual PE cases may not have been recorded, because they were detected as incidental findings in other radiology studies conducted for different clinical reasons, such as contrast-enhanced chest or abdominal CT exams. For study feasibility and consistency reasons, we decided to not include such incidental PE findings in our analysis, because (i) the number of such cases is usually small, and (ii) the detection of incidental PE in other radiology studies will frequently trigger the subsequent acquisition of a dedicated CTPA study for further evaluation of this incidental finding, which would then eventually be captured by our list of descriptors for PE-related CTPA studies. Despite efforts to use institution-specific exam search terms that are specific to PE studies, there is the potential for erroneous inclusion of a small number of thoracic angiography studies that are not dedicated PE studies. This error would apply to both, the pre- and intra-pandemic time periods.

A single time point was chosen to mark the onset of the pandemic. This was done for practical reasons and despite minor variations in time with regard to the onset of the pandemic in different geographic areas of the country. The homogeneity of our observations suggests that this choice did not have a substantial impact on our findings. Another possible confounding factor to our results is the seasonal variation in pulmonary embolism incidence. Prior investigations have been mixed, with some studies showing no variation^45^ and other studies showing a peak in the fall and winter^46,47^. Measuring seasonal variation is a potential future application of the MIDH approach.

The number of participating healthcare systems was limited to centers able to participate based on their technological and informatics equipment and availability at the time of the investigation. Given the geographical distribution of the participating centers as well as a large number of analyzed cases, we believe that the findings are a representative reflection of the North American experience during the early period of the pandemic.

As an outlook for future research endeavors, we note that we did not retrieve additional detailed medical patient-specific information, such as patients’ COVID-19 status or underlying co-morbidities. For this reason, we have limited control over confounding variables, because we may not be able to ascertain whether observed differences in the PE prevalence between the two observation periods may be attributed to patients’ specific medical or other non-retrieved information. Although there is no technical obstacle to retrieving such information from healthcare information systems, such large-scale extraction of personal health information involves significant challenges regarding IT security and HIPPA compliance within a multi-centric study, involving multiple healthcare systems with different technical, administrative, and legal infrastructures, including varying guidelines for granting IRB approval for such studies. Despite these challenges, it is clear that more granular medical data would provide significant opportunities for future study in this domain.

To place our scientific contribution into the context of the current state-of-the-art in digital medicine, it should be mentioned that methods for mining electronic health records, radiology information systems, or other IT systems for measuring imaging utilization and defining the presence/absence of disease conditions have been well-studied in the field of radiology^48^. However, using the data of single institutions may not provide a sufficient number of cases related to a specific disease entity, which limits the statistical power of such studies, regardless of the high number of originally screened radiology reports. For example, several of the participating institutions in our study were capable of mining millions of their own radiology reports using commercially available NLP tools with regard to the presence of pulmonary embolism on CTPA. Yet, as can be seen from Supplementary Fig. 1, none of the participating individual institutions would have been able to provide a sufficient number of cases to unambiguously infer a statistically significantly increased observed PE prevalence in CTPA exams during the early COVID-19 pandemic. Here, choosing the proposed multi-institutional data harvesting approach based on repurposed AI orchestration software can successfully address this challenge by significantly increasing the number of pertinent cases for the research question at hand, thus improving the statistical power of such observational studies.

In conclusion, we have discussed a multi-institutional data harvesting (MIDH) method for the longitudinal observation of medical imaging utilization and reporting based on repurposed AI-based workflow orchestration software. By tracking both large-scale utilization *and* clinical imaging results data, the MIDH approach is designed to establish surrogates for measuring important disease-related observational quantities over time, such as the observed prevalence of pulmonary embolism on CTPA exams during the early COVID-19 pandemic outbreak. Here, our retrospective multicenter study clearly documents an increase in the observed prevalence of PE on CTPA examinations during the early pandemic phase, despite an overall decrease in the number of acquired CTPA examinations, already before PE was recognized and established as a life-threatening complication of COVID-19 in the medical literature. As our retrospective analysis actually demonstrated an increased observed prevalence of pulmonary embolism during the early COVID-19 pandemic outbreak, it may be speculated whether MIDH-based “real-time” longitudinal data monitoring might have provided early clinical insight into the possible association between COVID-19 and thromboembolic complications long before such association was established in the medical literature and whether this digital medicine approach may therefore be useful for clinically meaningful decision making when facing future healthcare challenges with significant uncertainties, such as future pandemics.

## Methods

### Case selection

**Fig. 5:**
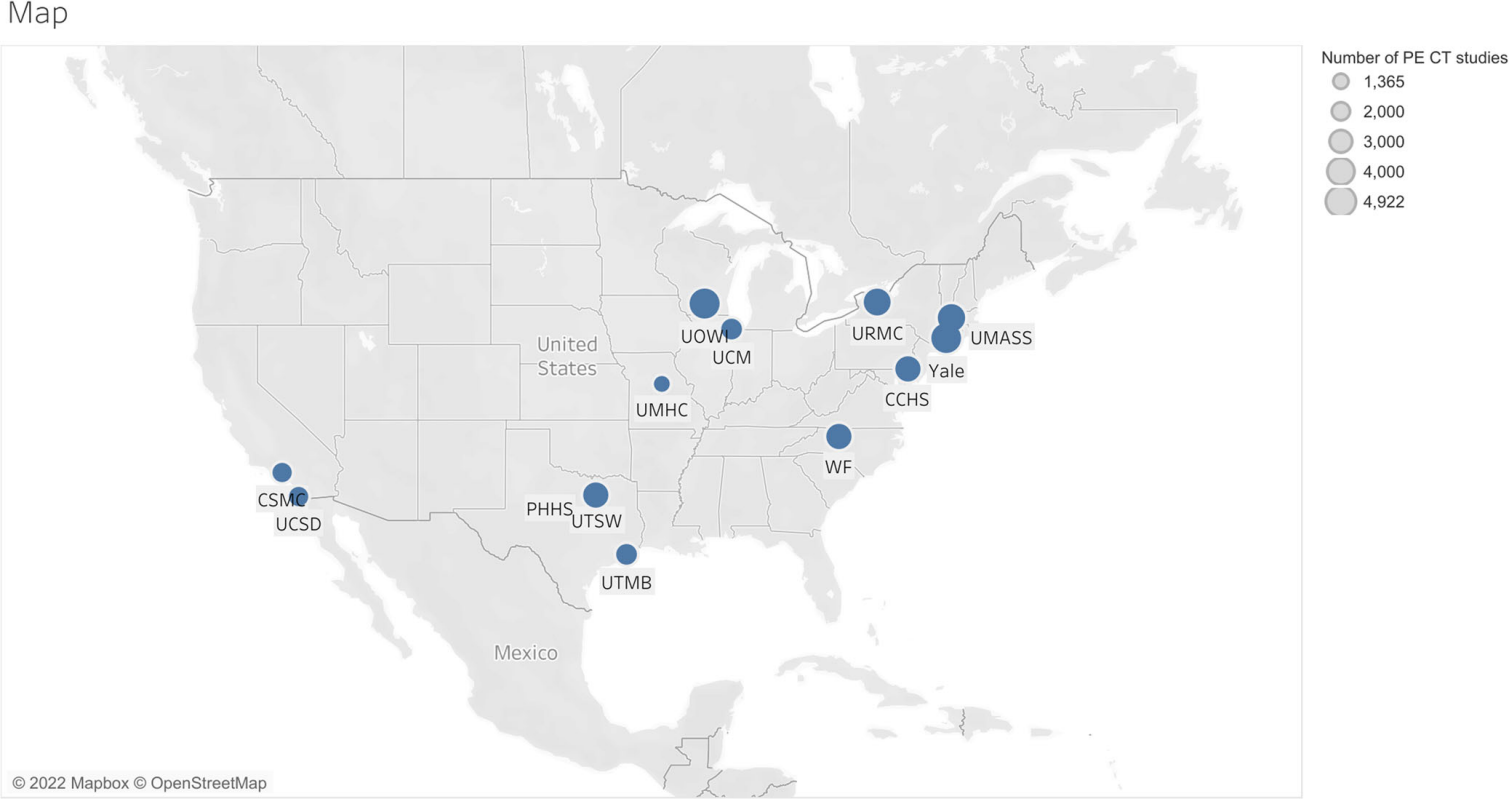
Geographic distribution of the participating institutions. CCHS Christiana Care Health System, CSMC Cedars-Sinai Medical Center, PHHS Parkland Health and Hospital System, UCM University of Chicago, UCSD University of California, San Diego, UMASS University of Massachusetts, UMHC University of Missouri-Columbia, UOWI University of Wisconsin-Madison, URMC University of Rochester Medical Center, UTMB University of Texas-Medical Branch, UTSW University of Texas-Southwestern, WF Wake Forest School of Medicine. The size of the circle is proportional to the number of cases contributed to this study by that institution. The detailed information by the site is available online (https://www.aidoc.com/resources/research/). Base map © OpenStreetMap (https://www.openstreetmap.org/copyright).

To investigate the clinical applicability of the MIDH approach for estimating the observed prevalence of pulmonary embolism on CTPA exams, we retrospectively collected data on CTPA exams performed within 13 healthcare systems throughout the United States (Fig. 5) and associated radiology reports. The number of cases provided by each participating site is provided in Supplemental Materials (Supplementary Fig. 1). Data were collected using software that was originally developed for data workflow orchestration by a commercial AI-based image analysis service (Aidoc, Tel Aviv, Israel). This service is typically used to perform AI-based reprioritization of radiologists’ reading worklists with the goal of decreasing radiology study turn-around-time, thereby expediting treatment in critical clinical conditions, such as intracranial hemorrhage or pulmonary embolism^34,49^. However, for this study, we did not use any AI-based image analysis, but only used the underlying data workflow prioritization software to automatically retrieve CTPA cases within the participating healthcare systems. Specifically, the repurposed data workflow prioritization solution was based on a robust study identification mechanism that relied on a minimal set of pre-defined metadata terms, such as institutional-specific study description keywords, see Table 4 for details. In a first step, these terms were used to automatically select CTPA studies. In a second step, these identified CTPA studies were automatically checked against inclusion criteria, see details in section “Data collection” below, and the final radiology reports associated with the selected CTPA studies were automatically retrieved. In a third step, these radiology reports were automatically classified for the presence or absence of PE by using natural language processing (NLP), see details in section ‘Radiology report classification’ below. Note that, with the exception of institution-specific pre-defined metadata terms for case selection as specified in Table 4, all software components used for these processing steps were identical across all 13 participating healthcare systems.

**Table 4:** Inclusion criteria for CTPA studies at each institution.

Institution	Inclusion criteria
1. CCHS	Any study description that included “PE”
2. CSMC	Any protocol name that included “PE”/“P.E”/“Pulmonary embolism”
3. PHHS	Any study description that included “Angio* Chest”
4. UCM	Any study description that included “PE”/“Pulmonary embolism”
5. UCSD	All cases with the study description “CTA PULMONARY EMBOLUS”
6. UMass	Any procedure description that included “PE”/“Pulmonary emb”/“ CT ANGIOGRAM CHEST W”
7. UMHC	Any study description that included “PE”/“CT pulmonary angiogram”
8. URMC	All cases with the procedure description “CT ANGIO CHEST”
9. UTMB	Any procedure description that included “PE”/“Pulmonary emb”/“Angio* Chest”
10. UTSW	Any study description that included “Angio* Chest”
11. UOWI	Any procedure description that included “PE”/“P.E.”/“Pulmonary emb”/“Angio* Chest”/“CTA CHEST”
12. Wake Forest	Any procedure description that included “PE”
13. Yale	Any study description that included “PE”/“Pulmonary emb”

The case retrieval rates for the purpose of the research were set as a background task with rates between 100 and 500 cases per day per site, which was dependent on a number of institutional factors, such as server and network loads. The process could be scaled as required. The data processing failures were under 0.05% for the purpose of this study. The main reason for failure were incomplete data, which was rare as only textual data were used.

While other technical methods for retrieving CTPA datasets, such as based on electronic medical records, radiology information systems, or Picture Archiving and Communication Systems (PACS), might have been considered from a technical viewpoint, our approach using repurposed AI-image-analysis orchestration software provided the advantage of being a common system, already deployed in clinical routine, with an already validated and robust study identification mechanism, which provided easy access and consistent data collection across multiple institutions with different individual technical infrastructures.

### Data collection

We compiled 40,037 CTPA studies from 13 participating US healthcare systems acquired from 11/1/2019 through 6/30/2020. This observation period was chosen based on the time course of the pandemic and to reflect an extended time period of stable IT systems operation across all participating institutions without major technical changes, such as product transitions or major software updates.

Two 70-day observational periods were compared:

(i) the pre-pandemic period from 11/25/2019 through 2/2/2020, and
(ii) the early COVID-19 pandemic period from 3/8/2020 through 5/16/2020.

While the first case in the United States was confirmed in Seattle on 1/20/2020^50^, it was not until 2/26/2020 that the first non-travel associated case of COVID-19 was confirmed in California^51^. Therefore, the 70 days of PE reports included in the pre-pandemic cohort occurred prior to the detection of the virus in any state included in this study. In most of the participating sites, a significant reduction in CTPA volume occurred on 3/8/2020, and COVID-19 was labeled a pandemic by the World Health Organization just 3 days later on 3/11/2020. The time period between the pre-pandemic and the early pandemic periods (2/2/2020–3/7/2020) was excluded in order to decrease the effect of patients that presented before the pandemic was fully appreciated.

We automatically retrieved all CTPA exams based on procedure description. Based on technical and administrative differences among the participating healthcare systems, the study descriptors used varied slightly across institutions. A detailed list of study descriptors for each institution is shown in Table 4. Patients under the age of 18 were excluded and all patients over the age of 90 were considered to be 90. Patients of unknown age, which comprised ~10% of the total cohort (4192 of the 40,037 collected studies patients), were included in the analysis. One of the sites (Yale) had incomplete age data due to a technical issue in passing birth dates to Aidoc. The site corrected this problem during the study period.

The number of CTPA exams and the number of positively reported cases based on NLP were recorded. For each retrieved CTPA study, the patient’s age, sex, and patient location (emergency, inpatient, outpatient, other) were recorded. Only anonymized aggregated data was shared among the consortium, whereas patient-specific medical data, such as COVID-19 testing results or underlying co-morbidities, were not collected or used for this study.

### Radiology report classification using NLP and NLP validation

An NLP tool (“RepScheme”, Aidoc, Tel Aviv, Israel) was used to classify studies as positive or negative for PE, according to the report text. The rule-based NLP tool allows the classification of radiology reports according to the presence of specific pathologies in conjunction with advanced textual analysis. It is based on expert-designed queries, composed of radiology and clinical terminology building blocks (such as “thrombus” and “emboli”), connected using logical structures (such as AND, OR, positive context, negative context). Other NLP techniques used by the system include negation detection based on dependency parsing^52^. A diagram describing the specification rules is provided in the Supplementary Material (Supplementary Fig. 2).

As part of the study, the NLP performance was validated in 12 of the participating institutions. For each institution, a subset of 100 randomly selected PE-positive and 100 randomly selected PE negative cases as determined by the NLP, termed “PE + NLP” and “PE − NLP”, respectively, were blindly reviewed by radiologists (attending or resident level) and classified as being positive for PE (“PE + manual”) or negative for PE (“PE − manual”) to determine NLP accuracy. The review process was “blind” in the sense that reviewers did not use radiology images, but only radiology reports. Note that the task performed by the reviewers was markedly simpler than reading radiology studies, because it was restricted to solely classifying radiology reports for the presence or absence of reported PE. The manual review results of the radiology reports served as the ground truth for calculating NLP accuracy rates. As the NLP configuration used for the study was focused on the detection of acute PE, cases were considered negative if only chronic thromboembolic disease was present. Studies that were classified indeterminate for PE based on the report were considered negative.

### Statistical analysis

Demographic data for the two observation periods were compared using the Wilcoxon rank-sum test for age and the χ^2^ test for the other categorical variables. The ratio of exams that were positive for PE were compared between the pre-COVID-19 and early COVID-19 pandemic observational periods, and a χ^2^ test of independence was performed to assess the association. These tests were performed using Stata version 13.1 (StataCorp, College Station, Texas).

Logistic regressions were used to estimate the odds ratio of positive PE findings between the pre-pandemic and the early pandemic periods for each site individually. Patient location and gender were included as covariates. A generalized estimating equation (GEE) was used to combine the estimated COVID-19 effect from all sites. Clustering of exams within sites was adjusted by a site-specific random effect. Marginal estimation of the PE positivity rates from the pre- and early- pandemic periods were also reported. Further, the interaction terms between observational periods and each of the covariates were specifically tested. For example, a significant interaction term between gender and observational periods would indicate differences in PE positivity rates between males and females between the pre-COVID-19 and the early COVID-19 pandemic periods. 10% of the entire cohort, was not used as a covariate in the initial multivariable analysis. A sensitivity analysis was carried out by adding age as a covariate to the multivariable GEE model, including only patients with known age ≥18 years. The results are reported in the Supplemental Materials. Statistical analysis was performed using SAS version 9.4 (SAS Institute Inc., Cary, NC). *P* values < 0.05 were considered statistically significant.

### Ethics

Formal institutional review board approval was acquired by the local ethics committee at each participating institution, including Christiana Care Health System, Cedars-Sinai Medical Center, Parkland Health and Hospital System, University of Chicago, University of California, San Diego, University of Massachusetts, University of Missouri-Columbia, University of Wisconsin-Madison, University of Rochester Medical Center, University of Texas-Medical Branch, University of Texas-Southwestern, and Wake Forest School of Medicine. The requirement for informed consent was waived. Project identification numbers are provided in Table 5.

**Table 5:** Individual institutional review board study numbers.

Institution name	IRB study identification
UCM	IRB19-0804-CR001
CCHS	40109
UTSW	STU-2020-0818
Yale	2000027425
UMHC	2017710
CSMC	STUDY00000897
UCSD	201436
UMass	H00020868
Wake Forest	IRB00068157
PHHS	STU-2020-0818
UTMB	20-0176
URMC	STUDY00005185
UOWI	2016-0418

### Reporting summary

Further information on research design is available in the Nature Research Reporting Summary linked to this article.

## Supplementary information

Supplemental Material

Reporting Summary

## Data Availability

The datasets generated and analyzed in this study are available on request from the corresponding author [A.S.B.]. The original radiology data were not publicly available, because they represent patients’ protected health information.

## Code availability

Export and analysis code is available upon request.
