# Supplementary material for: Early-stage COVID-19 pandemic observations on pulmonary embolism using nationwide multi-institutional data harvesting
Source: NPJ Digit Med. 2022 Aug 19;5:120. doi: 10.1038/s41746-022-00653-2 (PMC9388980; doi:10.1038/s41746-022-00653-2)

**Supplemental Material**

**
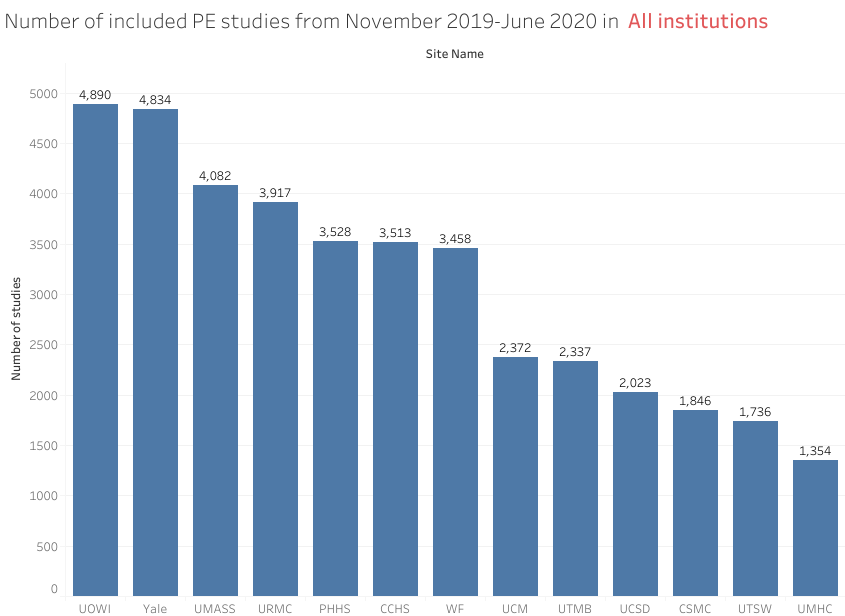
Supplementary Figure 1: Number of cases provided by each participating site.** CCHS=Christiana Care Health System, CSMC=Cedars-Sinai Medical Center, PHHS=Parkland Health and Hospital System, UCM=University of Chicago, UCSD=University of California, San Diego, UMASS=University of Massachusetts, UMHC=University of Missouri-Columbia, UOWI=University of Wisconsin-Madison, URMC=University of Rochester Medical Center, UTMB= University of Texas-Medical Branch, UTSW=University of Texas-Southwestern, WF= Wake Forest School of Medicine.


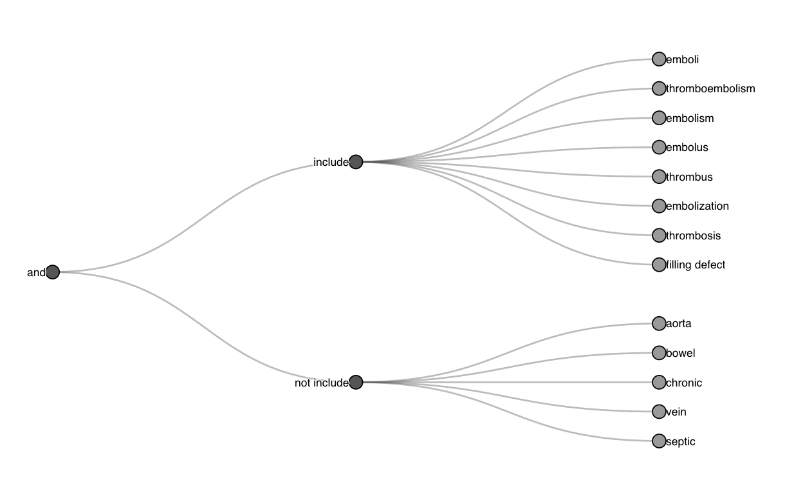


**Supplementary Figure 2**: **The specification of the rule-based component of the NLP algorithm.**

**
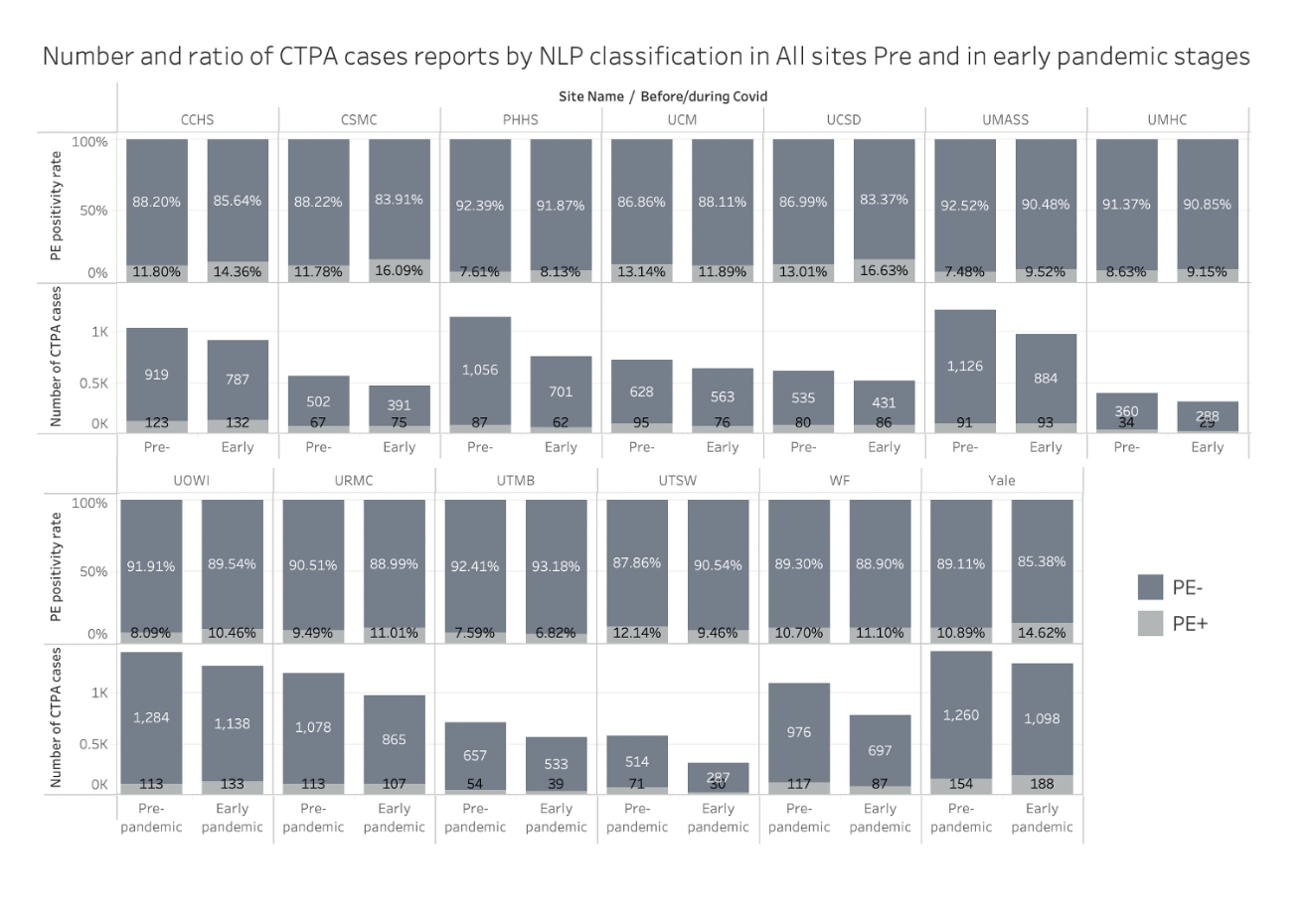
 Supplementary Figure 3: Acquired CTPA exams in each institution.** Total number of acquired CTPA scans and percentages of PE+ studies for individual institutions during the observation periods before and during the early COVID-19 pandemic. It is clearly seen that the number of CTPA studies decreased for all institutions, while the percentage of PE+ studies increased during the early COVID-19 pandemic outbreak for most institutions.

**Supplementary Table 1: Results of manual review of the NLP performance at each individual site.**

| **CCHS** | **Manual classification** | |  |
| --- | --- | --- | --- |
| **NLP classification** | **PE -** | **PE +** | **Total** |
| **PE -** | 98 | 2 | 100 |
| **PE +** | 6 | 94 | 100 |
| **Total** | 104 | 96 | 200 |
| **CSMC** | **Manual classification** | |  |
| **NLP classification** | **PE -** | **PE +** | **Total** |
| **PE -** | 99 | 1 | 100 |
| **PE +** | 2 | 98 | 100 |
| **Total** | 101 | 99 | 200 |
| **UCM** | **Manual classification** | |  |
| **NLP classification** | **PE -** | **PE +** | **Total** |
| **PE -** | 100 | 0 | 100 |
| **PE +** | 0 | 100 | 100 |
| **Total** | 100 | 100 | 200 |
| **UCSD** | **Manual classification** | |  |
| **NLP classification** | **PE -** | **PE +** | **Total** |
| **PE -** | 99 | 1 | 100 |
| **PE +** | 7 | 93 | 100 |
| **Total** | 106 | 94 | 200 |
| **UMASS** | **Manual classification** | |  |
| **NLP classification** | **PE -** | **PE +** | **Total** |
| **PE -** | 100 | 0 | 100 |
| **PE +** | 8 | 92 | 100 |
| **Total** | 108 | 92 | 200 |
| **UMHC** | **Manual classification** | |  |
| **NLP classification** | **PE -** | **PE +** | **Total** |
| **PE -** | 100 | 0 | 100 |
| **PE +** | 1 | 99 | 100 |
| **Total** | 101 | 99 | 200 |
| **UOWI** | **Manual classification** | |  |
| **NLP classification** | **PE -** | **PE +** | **Total** |
| **PE -** | 100 | 0 | 100 |
| **PE +** | 0 | 100 | 100 |
| **Total** | 100 | 100 | 200 |
| **URMC** | **Manual classification** | |  |
| **NLP classification** | **PE -** | **PE +** | **Total** |
| **PE -** | 100 | 0 | 100 |
| **PE +** | 9 | 91 | 100 |
| **Total** | 109 | 91 | 200 |
| **UTMB** | **Manual classification** | |  |
| **NLP classification** | **PE -** | **PE +** | **Total** |
| **PE -** | 97 | 3 | 100 |
| **PE +** | 0 | 100 | 100 |
| **Total** | 97 | 103 | 200 |
| **UTSW** | **Manual classification** | |  |
| **NLP classification** | **PE -** | **PE +** | **Total** |
| **PE -** | 98 | 2 | 100 |
| **PE +** | 1 | 99 | 100 |
| **Total** | 99 | 101 | 200 |
| **Yale** | **Manual classification** | |  |
| **NLP classification** | **PE -** | **PE +** | **Total** |
| **PE -** | 100 | 0 | 100 |
| **PE +** | 3 | 97 | 100 |
| **Total** | 103 | 97 | 200 |
| **WF** | **Manual classification** | |  |
| **NLP classification** | **PE -** | **PE +** | **Total** |
| **PE -** | 97 | 3 | 100 |
| **PE +** | 1 | 99 | 100 |
| **Total** | 98 | 102 | 200 |

**Sensitivity Analysis including Age**

After adjusting for patient class, gender, and age, the estimated overall OR was 1.19 (95% CI 1.07-1.32, *p*=0.02). The adjusted PE positivity rate was 9.5% (8.2%-11.0%) in the control period and 11.1% (9.6%-13.0%) in the early COVID-19 period. None of the interaction terms between COVID-19 and the covariates were statistically significant at the 0.05 level (*p*=0.58 (patient class*COVID-19), 0.22 (gender*COVID-19), and 0.54 (age*COVID-19)), demonstrating that the effect on patient class and gender on PE positivity was consistent between the two observation periods.


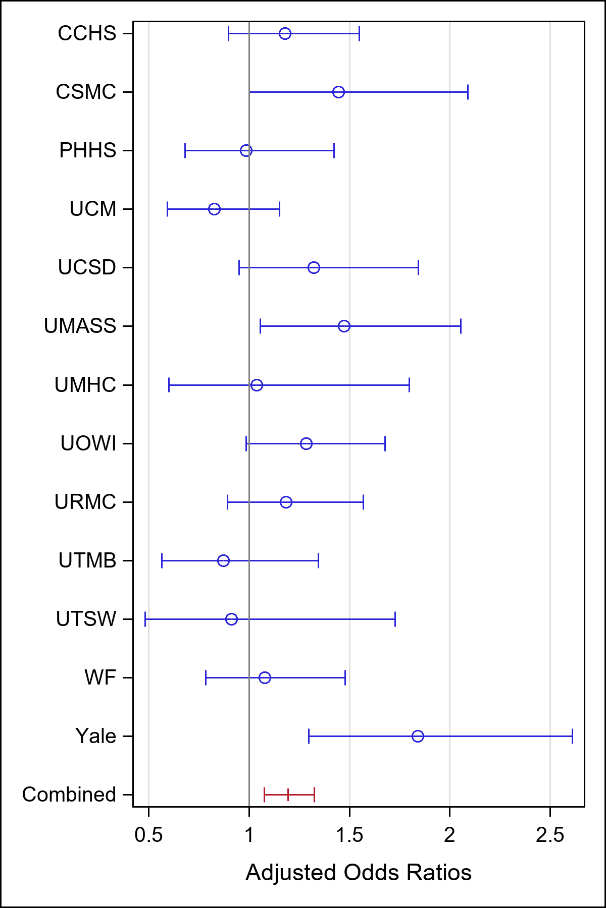

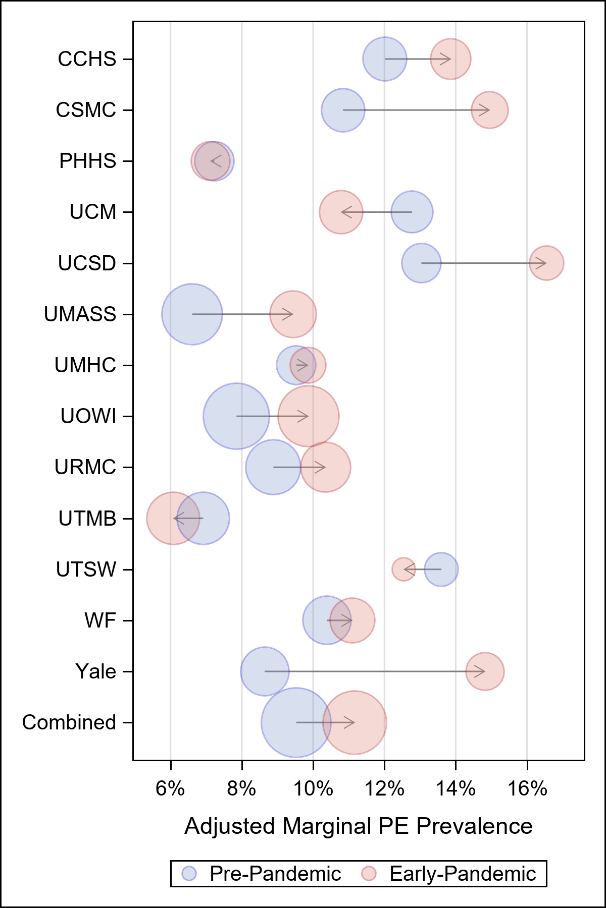

Supplement: Supplementary file 1 — Supplemental Material [file 41746_2022_653_MOESM1_ESM.docx]
